# A general framework to model the fate of trace elements in anaerobic digestion environments

**DOI:** 10.1038/s41598-021-85403-2

**Published:** 2021-04-05

**Authors:** Bikash Chandra Maharaj, Maria Rosaria Mattei, Luigi Frunzo, Eric D. van Hullebusch, Giovanni Esposito

**Affiliations:** 1 Department of Civil and Mechanical Engineering, University of Cassino and the Southern Lazio, Via di Biasio 43, 03043 Cassino, FR Italy; 2 Department of Mathematics and Applications “Renato Caccioppoli”, University of Naples Federico II, Naples, Italy; 3 Department of Civil, Architectural and Environmental Engineering, University of Naples Federico II, Naples, Italy; 4 Institut de Physique du Globe de Paris, Université de Paris, CNRS, F-75005 Paris, France

**Keywords:** Environmental biotechnology, Bioenergy, Biogeochemistry, Applied mathematics

## Abstract

Due to the multiplicity of biogeochemical processes taking place in anaerobic digestion (AD) systems and limitations of the available analytical techniques, assessing the bioavailability of trace elements (TEs) is challenging. Determination of TE speciation can be facilitated by developing a mathematical model able to consider the physicochemical processes affecting TEs dynamics. A modeling framework based on anaerobic digestion model no 1 (ADM1) has been proposed to predict the biogeochemical fate TEs in AD environments. In particular, the model considers the TE adsorption–desorption reactions with biomass, inerts and mineral precipitates, as well as TE precipitation/dissolution, complexation reactions and biodegradation processes. The developed model was integrated numerically, and numerical simulations have been run to investigate the model behavior. The simulation scenarios predicted the effect of (i) organic matter concentration, (ii) initial TEs concentrations, (iii) initial Ca–Mg concentrations, (iv) initial EDTA concentration, and (v) change in TE binding site density, on cumulative methane production and TE speciation. Finally, experimental data from a real case continuous AD system have been compared to the model predictions. The results prove that this modelling framework can be applied to various AD operations and may also serve as a basis to develop a model-predictive TE dosing strategy.

## Introduction

Anaerobic digestion (AD) of organic wastes is a well-established alternative source of sustainable energy^1^. Efforts have been devoted to improve the biogas production by recruiting process optimization and scale up techniques^2–7^. Dosing of trace elements (TEs: Fe, Ni, Co, Se, Mn, Zn and Mo) in AD is one of such avenues^8–11^. This is primarily due to the sensitivity of the microbial community to TEs, in particular acetogens and methanogens. Indeed, the syntrophy between acetogens and methanogens is largely attributed to optimum availability and supply of TEs. Any lack or excess of TEs in the reactor leads to acidification and toxicity, respectively. Moreover, the complex biogeochemistry of TEs requires a thorough analysis regarding the fate of TEs in AD systems^12^. Examining the biogeochemistry and the dynamic behavior of TEs in AD is necessary to individuate important process parameters which would be the first step towards a model predictive TE dosage control.

TEs are structural components of important enzymes involved in AD biochemical processes^13^. Fe is a structural component of the majority of enzymes in AD pathways^14,15^. Also, it serves as a redox carrier^16^. Ni is a part of coenzyme F_430_ ring structure of methyl reductase which facilitates reductions of methyl coenzyme M to methane during methanogenesis^17^. Co is essential to both acetoclastic and hydrogenotrophic methanogens. It is an integral part of coronoid structure of vitamin B12 which binds to coenzyme methylase^18^. Thus, these bioavailable TEs are necessary micronutrients for the consortium of microorganisms present in AD.

The bioavailable fraction of TEs in an AD system is a function of the bio-uptake, precipitation, complexation and adsorption processes^19,20^. Proper bioavailability of TEs in an AD system is necessary for an efficient methane production^21^. Microbial growth depends on the uptake and transport of these essential TEs from the bulk solution^19^. Quantification of mineral precipitation/dissolution^22–30^, organic complexation, and adsorption/desorption is crucial to determine the TE bioavailability in the liquid phase.

Due to the significant role played by TEs in AD systems, the difficulty in measuring the bioavailable TE fractions^31,32^, as well as the intricate TE biogeochemistry, a mathematical model able to simulate the complex dynamics of TEs in anaerobic environments would be a useful tool^21^. Although there have been experimental efforts to quantify TE in the liquid phase of anaerobic reactors, a mathematical model-based approach will augment and accelerate the TE quantification towards an accurate and reliable dosing strategy designed on case to case basis.

In this regard, incorporating physicochemical processes in AD modelling is crucial. Many studies have higlighted the omission of important physicochemical processes (other than gas transfer and acid base transformation) in ADM1^33^. Subsequently, some attempts have been made to improve the physicochemical predictions of the model. In a minor model improvement calcium carbonate (CaCO_3_) precipitation was included to demonstrate an industrial application of the model^23^. Later on, a more detailed physicochemical framework was implemented to study the precipitation of phosphorous with alkaline earth metals (Ca and Mg)^27^. Similarly, the interactions of phosphorous, sulfur and iron were studied^25^ with emphasis on phosphorous recovery and iron precipitation as one of the processes affecting microbial kinetics. Evidently, these physicochemical improvements mainly focused on the reactions of phosphorous metabolism. Moreover, only selected precipitation/dissolution reactions have been considered by these models while neglecting other major physicochemical processes such as complexation and adsorption/desorption. Recently, a model for TE quantification has been proposed which is mechanistic in nature and considers only representative components and species^34^, thus lacking a detailed description of major species and components required to understand TE biogeochemistry in AD. Apart from these, there have been attempts to describe complexation^35^ and adsorption processes^36^ in wastewater systems but they are not specific to anaerobic environments. Ultimately, these studies would not be sufficient to understand the complex mechanisms associated with TEs in AD as, more often, they lack one or many components and processes necessary to describe the TE biogeochemistry in AD systems^26,37^.

In contrast, numerous scientific reports^20,27,33,38–41^ have highlighted the importance of considering multiple physicochemical processes (precipitation/dissolution, organic complexation, adsorption/desorption and ionic association dissociation), bio-uptake and components (Fe, Ni, Co, Ca, Mg, Se, Mo) as responsible for TE dynamics in AD. To the best of our knowledge, there is no AD mathematical model which considers TE dynamics and attempts to quantify the complete profile of TE speciation across different phases. Therefore, in order to accurately predict the dynamics of TEs in AD, inclusion of additional processes and variables is required to the ADM1 model.

The main objective of this work is to develop a modelling framework to: (1) mechanistically describe all the main biochemical and physicochemical processes involved in an AD system, including adsorption of TEs (on inerts, biomass and precipitates), EDTA/VFA complexation, precipitation/dissolution and uptake of TEs; (2) investigate the speciation of TEs in AD, in particular the partitioning of TEs among adsorbed species, precipitates and dissolved complexes in relation to the change in initial concentration of complex organic matter, TEs, calcium, magnesium and EDTA. To this aim, new TE-adsorption reactions have been defined and added to the ADM1-based model developed^37^. Hydrolysis, acidogenesis, acetogenesis, methanogenesis and acid-base equilibrium have been combined to TE-adsorption, complexation and precipitation/dissolution and the effects on total methane production have been evaluated. Further, experimental data from a real case study have been compared with the model predictions in continuous mode of operation.

## Results

The implementation of the developed mathematical model has involved: (i) the update of the biochemical framework to include the effect of TEs on microbial kinetics as well as the release of sulfur, phosphorous and TEs during the disintegration step; (ii) the introduction of new acid–base reactions; (iii) inclusion of new liquid–liquid processes (TE-EDTA and TE-VFA complexation); (iv) the inclusion of solid–liquid processes (precipitation and dissolution); (v) incorporation of dynamic state variables and processes for sorption/desorption processes; (vi) the collection of the parameters values governing the sorption processes. In particular, these values have been collected from suitable literature and scientific reports^42^.

The capability of the model to cover a wide range of input variables and parameters is crucial to model applicability. In this regard, five different simulation scenarios (Table 1) have been considered to check the model prediction capability. *Scenario 1* analysed the impact of an increase in adsorbent concentration (complex organic matter) when the amount of TEs in the system is constant. *Scenario 2* evaluated the change in speciation of TEs when the initial concentration of a single TE is changed. Here the model simulations were performed for Fe, Ni and Co. *Scenario 3* examined any possible competition among carbonate and sulfide systems to capture mobile TEs onto available surfaces. *Scenario 4* was used to assess the response of the sorption reactions to the presence of a synthetic chelating agent. Lastly, *Scenario 5* showcased the variation in the model prediction due to change in binding site density for biomass, inerts and precipitates. In addition to *in-silico* experiments, a continuous mode of operation was implemented to compare model behaviour with a real case study demonstrating the effect of TE starvation on methane production.

### Scenario 1: Effect of adsorbents on TE dynamics in anaerobic digestion

**Figure 1:**
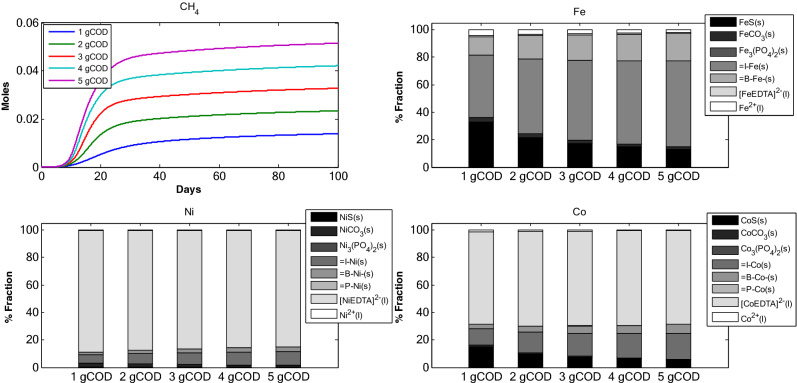
TE speciation and methane production in an anaerobic batch reactor, under different initial complex organic matter concentrations.

*Scenario 1* assesses the change in TE sorption with increase in $X_{c}$ amount in the reactor with $B_{\delta } = 2 \times 10^{ - 3}$, $I_{\delta }$ = $2 \times 10^{ - 3}$ and $P_{\delta }$ = $2 \times 10^{ - 5}$ moles/g of dry weight. $B_{\delta }$ is the biomass binding site density, $I_{\delta }$ is the inert binding site density and $P_{\delta }$ is the precipitate binding site density. The initial TEs concentration and other initial concentrations were kept constant **(**Tables 1, 2). What stands out in this scenario is the increase in adsorption of TEs to inert with increase in initial $X_{c}$ (Fig. 1). This is more pronounced in Fe fractions where I–Fe_(s)_ increases from around 45% of Fe to about 70% of Fe for corresponding initial $X_{c}$. Ni and Co also share similar behaviour with inert. Consequently, the fraction for TE-EDTA decreased with increase in initial $X_{c}$. Similar is the case for biomass fraction, B-TE_(s)_. Further, with the increase in initial $X_{c}$, the sulfide precipitate decreased. Sulfide fraction for Fe was higher than Ni and Co for corresponding initial $X_{c}$. The majority of Ni and Co (< 70%) was found associated to EDTA. For Ni and Co, the TE-EDTA fraction remained more or less constant across all initial $X_{c}$ concentrations (1–5 gCOD of composite organic matter). It is interesting to note (Tables 1, 2) that for all model runs, the cumulative methane production (Fig. 1) increased. This may be due to increased amount of organic matter and an increased TEs release into the system which is proportional to $X_{c}$.

### Scenario 2: Effect of initial Fe, Ni and Co concentration on TE dynamics in anaerobic digestion

**Figure 2:**
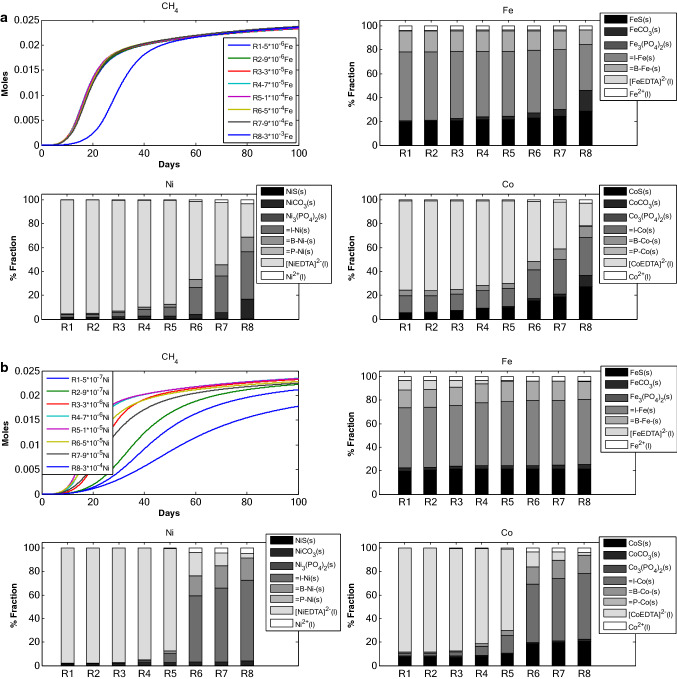

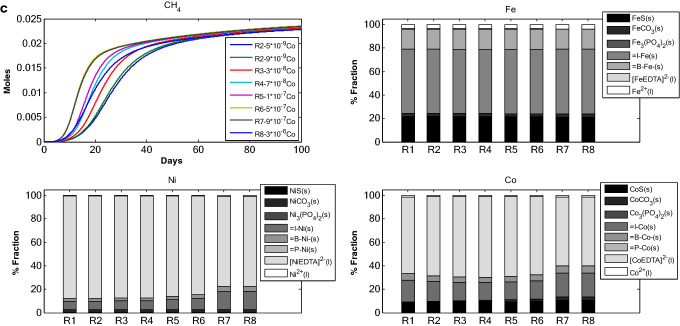
(**a**) TE speciation and methane production in an anaerobic batch reactor, under different initial Fe concentrations. (**b**) TE speciation and methane production in an anaerobic batch reactor, under different initial Ni concentrations. (**c**) TE speciation and methane production in an anaerobic batch reactor, under different initial Co concentrations.

*Scenario 2* studies the change in TE dynamics due to change in initial concentrations of TEs. Model simulation for 8 different initial concentrations for each TE (Fe, Ni and Co) was carried out (Fig. 2a–c). The initial concentration for the TE under study was varied while the other two TEs concentrations were set at an optimal value (Tables 1, 2).

#### Change in initial $S_{{Fe^{2 + } }}$ concentration

The amount of Fe adsorbed on inert matter is found to be higher at both lower and higher initial concentration of $S_{{Fe^{2 + } }}$(Fig. 2a). In RUN 1 through RUN 8, inert fraction of TEs is higher for Fe. Ni and Co have comparatively lower inert fraction, which increases rapidly in comparison to Fe inert fraction. Change in initial concentration of $S_{{Fe^{2 + } }}$ resulted in higher sulfide fraction for Fe and Co as compared to Ni. Ni and Co were found more with EDTA complex fraction. Additionally, with increase in initial $S_{{Fe^{2 + } }}$ concentration the carbonate fraction increases. The carbonate fraction for RUN 8 (with highest initial $S_{{Fe^{2 + } }}$ concentration, 3 × 10^–3^ M) is the highest among all the runs irrespective of the TE fraction. It is also interesting to note that complexation fraction is only observed in case of Ni and Co which decreases with increase in $S_{{Fe^{2 + } }}$ concentration. The free TE fraction for Ni and Co increases slowly with increase in $S_{{Fe^{2 + } }}$ initial concentration but is found to be constant for Fe. The cumulative CH_4_ production profile differs only for RUN 8 which corresponds to the highest $S_{{Fe^{2 + } }}$ concentration.

#### Change in initial $S_{{Ni^{2 + } }}$ concentration

TE-inert fraction is predominant in Fe for all initial concentrations of $S_{{Ni^{2 + } }}$ (Fig. 2b). However, for Ni and Co fractions, EDTA complex fraction is higher at lower initial concentration of $S_{{Ni^{2 + } }}$. With increase in initial $S_{{Ni^{2 + } }}$ concentration, the inert fraction increases for Ni and Co. Sulfide fraction was more prominent for Fe and Co. In case of Fe speciation, the fraction for sulfide and inert remains more or less constant for all the values of $S_{{Ni^{2 + } }}$ initial concentration. A thorough analysis of the fractionation pattern shows that with increase in initial $S_{{Ni^{2 + } }}$ concentration, the carbonate fraction does not increase significantly for Fe and Ni. It is also interesting to note here that complexation fraction decreases with increase in $S_{{Ni^{2 + } }}$ initial concentration. For Fe, the complexation fraction decreases with higher $S_{{Ni^{2 + } }}$ initial concentration. The free TE fraction changes with increase in $S_{{Ni^{2 + } }}$ initial concentration for Co and for Ni. In case of Fe, the free TE remained more or less constant throughout all the simulations. RUN 5 showed the highest cumulative methane production. Subsequently, the methane production decreased with further increase in initial $S_{{Ni^{2 + } }}$ concentration. The cumulative methane production for RUN 1, with 5 × 10^–7^ M of Ni, is around 2.3 × 10^–2^ M. For RUN 8 methane production is the lowest among all simulation runs.

#### *Change in initial*$S_{{Co^{2 + } }}$*concentration*

The trend of fractionation among Fe, Ni and Co is similar to the previous simulation sets for change in initial $S_{{Fe^{2 + } }}$ and $S_{{Ni^{2 + } }}$ concentrations (Fig. 2c). Higher amount of Fe was found adsorbed on inert matter at all initial concentration of $S_{{Co^{2 + } }}$. Comparatively, higher amount of sulfide was observed in Fe and Co fractions, and EDTA complexation fraction was higher for Ni. A very minute fraction of complexation is observed in all model runs for Fe. The fractionation of Fe shows little or no change with change in initial $S_{{Co^{2 + } }}$ concentration. The carbonate fraction does not increase significantly for all the TEs. The free TE fraction does not change significantly for any of TEs. The cumulative methane production did not change drastically with change in initial $S_{{Co^{2 + } }}$ concentration. The RUNs (1–8) resulted in cumulative methane production close to around 2.25 × 10^–2^ M. However, there was a variation in the rate of methane production.

### Scenario 3: Effect of initial $S_{{Ca^{2 + } }}$ and $S_{{Mg^{2 + } }}$ concentration on TE dynamics in anaerobic digestion

**Figure 3:**
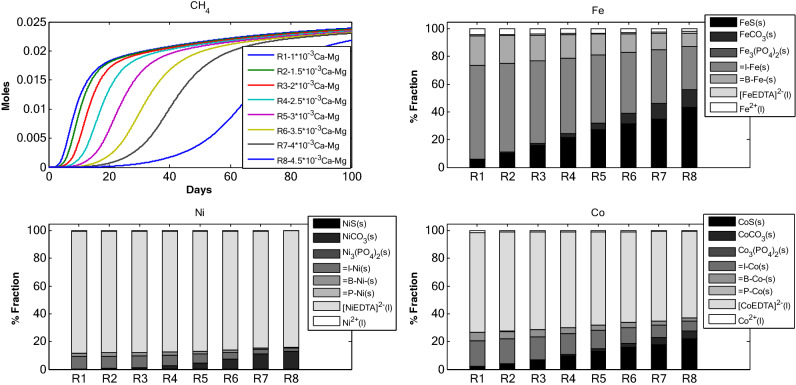
TE speciation and methane production in an anaerobic batch reactor, under different initial Ca–Mg concentrations.

*Scenario 3* investigates the effect of change in initial $S_{{Ca^{2 + } }}$ and $S_{{Mg^{2 + } }}$ concentrations on speciation of TEs and cumulative methane production (Fig. 3). With increase in $S_{{Ca^{2 + } }}$ and $S_{{Mg^{2 + } }}$ initial concentrations, inert and biomass fractions decreased for Fe, Ni and Co. For example, the amount of Fe bound to inert matter at lower initial concentration of $S_{{Ca^{2 + } }}$ and $S_{{Mg^{2 + } }}$ is higher in RUN 1 as compared to RUN 8. Conversely, amount of carbonate fraction for all the TEs increased with increase in $S_{{Ca^{2 + } }}$ and $S_{{Mg^{2 + } }}$ initial concentration. Free TE fraction was only noticed for Fe. For Ni and Co, the free TE fraction was close to zero and only appeared in the runs with lower initial concentration of $S_{{Ca^{2 + } }}$ and $S_{{Mg^{2 + } }}$. Sulfide fraction is principally found with Fe and increases for all TEs with increase in initial $S_{{Ca^{2 + } }}$ and $S_{{Mg^{2 + } }}$ concentration. EDTA complex fraction was found across all the simulations for Ni and Co. Fe was found associated more with inert fraction as well as biomass fraction with lower $S_{{Ca^{2 + } }}$ and $S_{{Mg^{2 + } }}$ concentration. With the increase in $S_{{Ca^{2 + } }}$ and $S_{{Mg^{2 + } }}$ initial concentration rate of cumulative CH_4_ production decreased, while the total amount of CH_4_ produced remained more or less constant for RUN 1–7 with a significant decrease for RUN8 which had highest amount of initial $S_{{Ca^{2 + } }}$ and $S_{{Mg^{2 + } }}$ concentration.

### Scenario 4: Effect of initial EDTA concentration on TE dynamics in anaerobic digestion

**Figure 4:**
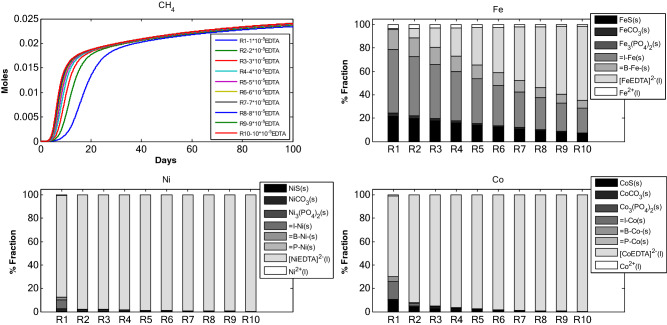
TE speciation and methane production in an anaerobic batch reactor, under different initial EDTA concentrations.

*Scenario 4* explored the effect of initial EDTA concentrations on the sorption of TEs and cumulative CH_4_ production with a set of 10 simulations (Fig. 4). The initial concentrations of Fe, Ni and Co are set at optimum values for all simulations (Tables 1, 2). The initial concentration of EDTA was varied from 1 × 10^–5^ M to 1 × 10^–4^ M (Tables 1, 2). Fe was found predominantly in inert fraction with lower initial concentration of EDTA. For example, the Fe-inert fraction is higher at RUN 1 as compared to RUN 10. As the initial concentration of EDTA increased, the Fe was observed more in the complexed fraction. However, Ni and Co were primarily found with complex fraction, even with lower initial EDTA concentration. It is interesting to note that, Fe is also found with sulfide fraction, whereas, Ni and Co is found with sulfide and inert fraction only at lower initial concentration of EDTA. The fraction of Fe bound to inert is higher as compared to the sulfide and complexed fraction for lower initial EDTA concentration. The free form of TEs decreased with increase in EDTA initial concentration. $S_{{Fe^{2 + } }}$ decreased with increase in EDTA initial concentration and a small fraction (~ 5% Fe) was found in the free form in RUN 10. However, Ni and Co free fractions were not observed in the model simulations other than RUN 1. Addition of higher amount of initial EDTA into the system did not significantly affect cumulative CH_4_ production. Nevertheless, the rate of methane production increased with higher initial EDTA concentration. The amount of methane produced at RUN 1 is close to 2.45 × 10^–2^ M and the amount of methane produced during RUN 10 is approximately 2.5 × 10^–2^ M.

### Scenario 5: Effect of binding site density on TE dynamics in anaerobic digestion

#### Change in biomass binding site density $B_{\delta }$

**Figure 5:**
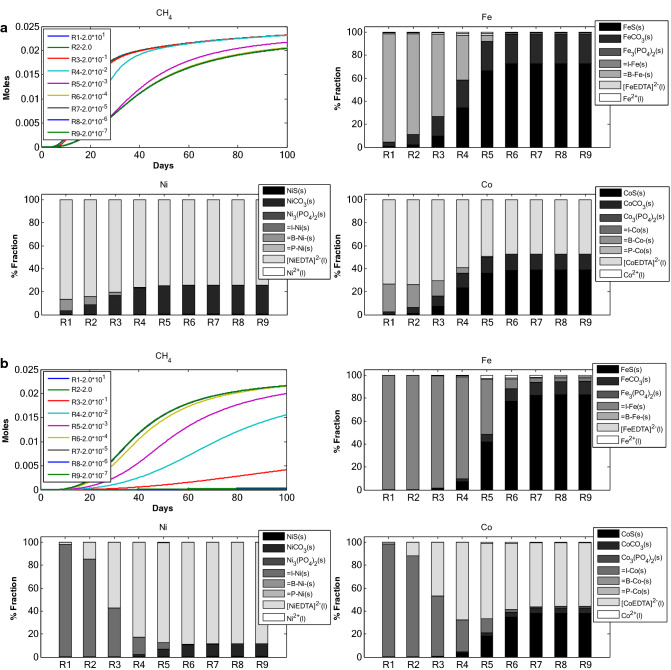

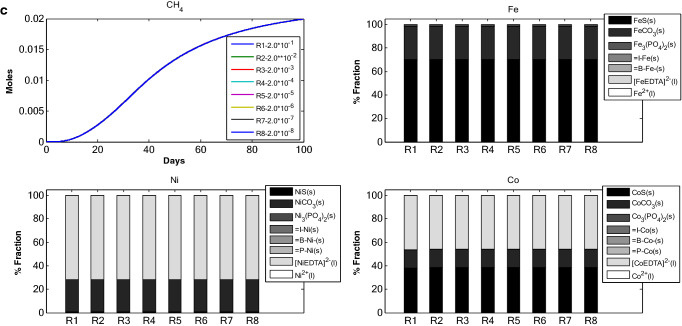
(**a**) TE speciation and methane production in an anaerobic batch reactor under different biomass binding site density. (**b**) TE speciation and methane production in an anaerobic batch reactor under different inert binding site density. (**c**) TE speciation and methane production in an anaerobic batch reactor, under different precipitate binding site density.

Nine simulations were carried out with different binding site density ($B_{\delta }$) for biomass (Fig. 5a, Tables 1, 2). The different binding site density was chosen while keeping in mind the binding site density reported in literature, which is 2 × 10^–3^ mol/g of biomass. With decrease in $B_{\delta }$ values, the fraction of Fe bound to biomass decreases (RUN 1 through 9, Fig. 5a). A similar trend has been observed for Co. This is also true for FeS and CoS. Change in $B_{\delta }$ values could affect the speciation of TEs but the distribution of TEs across different fractions remained more or less constant for RUN 5 through RUN 9. Maximum changes in free TE fraction were observed between RUN 1 and 5. With further decrease in $B_{\delta }$ values the speciation pattern did not change. Similarly, cumulative CH_4_ decreased till RUN 5 after which it remained constant.

#### Change in inert binding site density $I_{\delta }$

Figure 5b depicts 9 simulations carried out with 9 different $I_{\delta }$ values (Tables 1, 2). Change in $I_{\delta }$ altered the inert fraction for all model RUNs. The inert fraction for Fe decreased as $I_{\delta }$ decreased from RUN 1 to RUN 9. Whereas, the increase in binding site density for inert resulted in an increase in sulfide fraction. For Co and Ni similar speciation was observed but with two prominent characteristics. One, the free TE fraction decreased with decrease in inert binding site density for Ni and Co whereas in case of Fe, the free TE fraction increased up to RUN 5 and then decreased in RUN 9. Two, the EDTA complex fractions for Ni and Co were comparatively higher as compared to EDTA complexation fraction of Fe. The cumulative CH_4_ production was also examined (Fig. 5b) with change in $I_{\delta }$. RUN 9, with lowest inert binding site density, resulted in highest cumulative CH_4_ production.

#### Change in precipitate binding site density $P_{\delta }$

Figure 5c presents 8 simulations carried out with 8 different binding site densities for precipitates. In this model only FeS has been considered to harbour TE binding sites. The binding site density of FeS for Ni and Co has been assumed in this study (see “[Sec Sec13]”). The changes in binding site density of mineral precipitate did not result in change of TE speciation. The speciation for Fe, Ni and Co remains intact all throughout the RUNs from 1 to 8. Same is the case with cumulative methane production.

### Model comparison with a real case study

The model output has been qualitatively compared with the experimental results of a highly cited work^43^. The study assessed the impact of TE supplementation on single stage continuous mesophilic AD of food waste operated at bench scale. The substrate was a grounded mixture of fruits, vegetable, meat, fried foods and fat trimmings. A constant organic loading rate of 1.45 g/VSl/day was maintained for the reactors. The reactors were operated for 120 days at multiple HRT (25, 50, 100 and 180 (days)) with two reactors for each HRT. Each pair of such reactors was run with the same feedstock characteristics, but one of these reactors received TE supplementation periodically while the other one did not. The TEs have been dosed in a semi-continuous operation mode. The authors highlighted the failure of the reactors, after around 100 days, with no TE supplementation, whereas the reactors with TE supplementation exhibited a stable biogas production.

The starvation reactor with 50 days HRT was chosen for comparing the model output in terms of biogas production. The substrate characteristics and the operation parameters have been reported in Table 3. The model parameters, coefficients and stoichiometric values, have been selected based on the ADM1. Any change in the benchmarked model parameters have been explicitly mentioned in the text. The coefficients of the dose response function for a specific TE have been set at suitable values to capture starvation effect of TEs properly.

**Figure 6:**
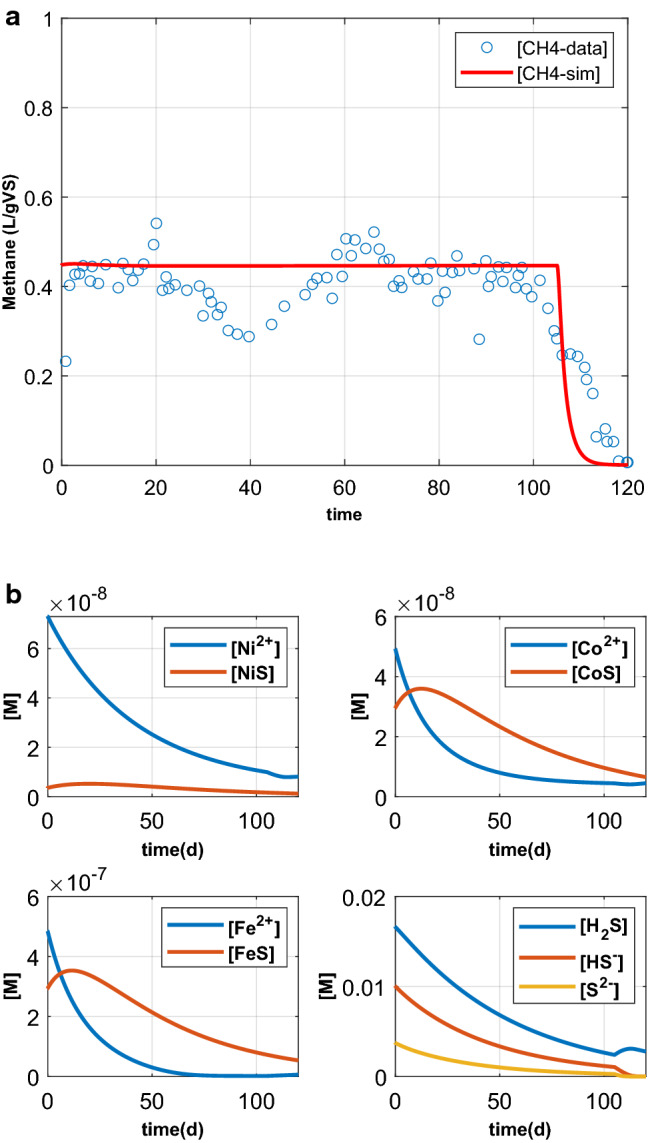
(**a**) Comparison of experimental data and model predictions for methane production of an AD reactor operated continuously with a 50 days HRT and food waste as feedstock. (**b**) Simulated dynamics of free TEs, precipitates and speciation of sulfide during model comparison with real case data.

The comparison of experimental data and model predictions for methane production has been summarized in Fig. 6a. According to the experimental results, TEs (Fe, Ni and Co) were the limiting factor which caused reactor failure after 100 days of operation. The methane production fell from apparently steady methane production of 0.43 L/gVS to 0 in the last 20 days of operation (Fig. 6a). The model has satisfactorily predicted the methane production during the AD of food waste. In particular, the model was able to reproduce the starvation effect of TEs on methane production in a continuous AD system. Furthermore, the free TEs along with speciation have been plotted in Fig. 6b, where TE depletion has been reported along with corresponding dynamics of sulfide precipitate in the system. Depletion of Ni, Co and Fe from the reactor have been the limiting factor which resulted in a reactor failure after around 100 days, which is the major finding of the experimental result^43^.

## Discussion

In an AD system, depending upon the reactor conditions (i.e. pH, OLR, redox state and hydraulic residence time), TEs are found in different chemical forms or species. The fate of TEs depend primarily on various biogeochemical processes occurring simultaneously at different rates. Precipitation, dissolution, organic complexation, adsorption, biotransformation of TE containing intermediates and bio-uptake are such determining processes. The resultant amount of free TE which eventually remains in the liquid phase depends on the amount of TEs involved in these mentioned biogeochemical fluxes. Precipitation/dissolution is a major flux which results in formation of sulfide, carbonate and phosphate minerals, and consequently scavenges the majority of TEs from the system. Similarly, organic complexation utilizes a portion of free TEs. TEs end up complexed with natural or synthetic organic ligands present in AD systems. A fraction of TEs is up-taken by biological cells and hence initiates other cascading processes (such as gene regulation, storage, excretion)^19,44^. However, some amount of TEs is supplied into the system by the biodegradation (in our case disintegration of complex organic matter) of TE containing intermediates. Adsorption/desorption of TE is an important and major physicochemical process, particularly when a large amount of surface area (biomass, inorganic precipitate and inert material) is available in the AD systems. The proposed model under discussion includes all the significant processes (including adsorption) involved in the speciation of TEs in AD. Apart from the standard biodegradation reactions (as in ADM1) the model includes 42 adsorption/desorption reactions, 13 precipitation/dissolution reactions and 15 complexation reactions. The adsorption of TEs has been considered on the biomass, precipitate and inert matter. The proposed model represents a general framework wherein the user/modeller can add and remove relevant inorganic components and species of interest as well as corresponding processes, to dynamically predict the TEs speciation. Such a customizable general model can be used as a precursor to build various model-based applications that aims at quantifying the fate of TEs in anaerobic digestion environments.

The proportional variations and dynamics of chemical fractions for adsorbed TEs under different initial concentration of: (i) adsorbents (Fig. 1), (ii) TE (Fe, Ni and Co, Fig. 2), Ca–Mg (Fig. 3), EDTA (Fig. 4) and the effect of change in binding site density (Fig. 5) have been studied. Throughout all the simulation scenarios, the adsorption fraction for inerts is higher than the adsorption fraction for biomass and precipitates. A possible explanation for this observation is that amount of inert material formed by the end of AD process is always higher as compared to the amount of biomass and mineral precipitate. Secondly, the % fraction is calculated at the end of the 100 days when the amount of biomass is already at lower limits and the inert materials reach maximum values. Thirdly, the binding site density may also influence the adsorption fraction**.**

Each simulation scenario is discussed individually below. Due to scarcity of literature we have tried to offer possible explanations based on theoretical understandings, first principles and our experience.

Change in initial concentration of $X_{c}$, as reported in Fig. 1, affects the TE adsorption fraction. The increase in TE adsorption fraction is more visible for inerts. There is no significant increase in adsorption to biomass and precipitate. This is primarily because with increase in $X_{c}$ the amount of inerts increases rapidly and accumulates in the system more efficiently as compared to biomass and precipitates. With increase in adsorbent concentration in the system the amount of adsorbed TEs increases (particularly with inert matter) for Fe, Ni and Co. This is because increase in adsorbent amount provides greater number of surface binding sites and hence the increase in adsorption. The decrease in free TEs can be attributed to the uptake of TEs by microbial communities as well as increase in adsorption flux.

The main objective of the scenarios with change in initial Fe (Fig. 2a), Ni (Fig. 2b)) and Co (Fig. 2c) was to predict the adsorption behaviour when limited and over supplied (in comparison to optimal concentration values) amount of TEs are present in the AD system. The simulation results indicate that the initial concentrations of Fe, Ni and Co have distinct patterns of effect on adsorption. Adsorption pattern as seen in case of different initial Fe concentrations (Fig. 2a) implies a decrease in adsorption fraction (for Fe and Co) with increase in TE dose. This may be explained by the fact that an increase in TE concentrations implies that TEs end up in sulfide fraction due to a suitable pH regime. Also, the difference in cumulative methane production for lower Fe concentration (RUN 1) and higher Fe concentration (RUN 8) is small because addition of more Fe does not affect the system. There may be two possible explanations: (a) as per our experience and literature anaerobic digestion system is more sensitive to change in Ni concentration; and (b) TE-EDTA complexes play a major role in stimulating methane production. However, this is not the case with change in initial Ni concentration (Fig. 2b) where a small change in initial concentration significantly affects the cumulative methane production. This may be because there is already enough free Ni in the system. Addition of a small amount of Ni into the system greatly affects the system both in terms of methane production and speciation of TEs. The increase in sulfide fraction and decrease in inert fraction implies an underlying mass transfer from inert fractions to the sulfide fraction. It can also be postulated that first the TEs get adsorbed on the surfaces and slowly get precipitated with increase in Ni dosing. Unlike Fe and Ni, change in initial Co concentration did not translate into any significant change in the TE speciation pattern (Fig. 2c). This may be attributed to the low concentration and high solubility of Co in the reactor system which can alter the reactor equilibrium significantly. A similar observation was made in a study^20^ which concluded an increased solubility of Co in presence of sulfide precipitates along with Fe and Ni. Interestingly, the model is able to capture small changes in methane production due to change in Co concentrations. This is an important aspect of the model, particularly when sensitivity of methane production to small concentration of TEs is considered.

Ca and Mg have been reported to exhibit competitive effect with TEs in terms of occupying binding sites. This is because of the similar charge of Ca and Mg as divalent TE ions. The decrease in methane production (Fig. 3) due to increase in Ca–Mg addition (Tables 1, 2), can be ascribed to the change in pH of the system and the availability of TEs. Moreover, the decrease in adsorption fraction with increase in Ca–Mg is due to increase in sulfide precipitate. At higher concentration of Ca–Mg, TEs precipitate with carbonates rather than adsorb on inert. This can be explained by the increase in saturation level of calcium–carbonate–magnesium systems due to increase in Ca–Mg concentration. This exercise confirms that with increase in TE dose the cumulative methane production first increases then decreases when the dose amount exceeds the optimal concentration limit. However, with increase in Ca–Mg dosage there is only decrease in cumulative methane production. This can partly be explained by the decrease in availability of free TEs at the higher concentration of Ca–Mg dosage.

Change in initial EDTA concentration, as reported in Fig. 4, affects the TE adsorption fraction. The decrease in TE adsorption fraction with addition of EDTA confirms its chelating ability. The inert fraction decreased along with decrease in sulfide precipitate fraction. The decrease in sulfide precipitate is in accordance to our experience^37^. The modelling framework presented here can be modified to take into account the chemistry of green chelating agents such as EDDS which has recently been investigated for its application and fate in anaerobic systems^41,45,46^.

Model application is not straightforward in the case of AD systems. Identifying correct inlet stream and operational parameters is crucial to get accurate model predictions. Consequently, model simulation depends on availability of data sets with all the necessary input characteristics, in particular the characterization of organic waste (particulate carbohydrate, proteins and lipids) in terms of COD and with regard to TEs. We selected a particular data set from published reports on TE supplementation in AD of food waste. The data set was selected by keeping in mind the specific effect of TEs (here starvation) it demonstrates in the continuous reactor operation.

Model calibration and validation is more complicated when physicochemical processes such as precipitation, complexation and adsorption are considered in addition to biodegradation processes. This is because the range of TEs concentration, in terms of free TEs, required for optimum microbial activity is rarely reported in conjugation with methane production and other major intermediates of AD process. Moreover, in most studies organic matter is characterized in terms of volatile solids content and the relationship between volatile solids content and COD varies for each specific substrate. Also, the characterization of organic waste in terms of content of sulfur, phosphorous and TEs is difficult to find for a particular study simultaneously. The primary reason for such a discrepancy is due to the variety of substrates used and the diversity of research objectives. Nevertheless, with the increasing amount of data in recent years dedicated to microbial community analysis in AD, the effect of TEs on methanogenesis and experimental speciation analysis, it would be relatively more convenient to calibrate and validate TE dynamics and speciation in AD system in the near future.

The recruitment of a model-based control strategy of TEs addition to AD system may require additional model complexity in terms of significant processes and components. Likewise, considering EPS (extracellular polymeric substances) as a storage house for complexation of TEs, which in turn affects the binding site density and hence adsorption behaviour, it is necessary to incorporate EPS and SMP (soluble microbial products) in order to achieve a more accurate mathematical model. Nevertheless, the goal of this study is to build a model framework based on ADM1 which can simulate the effect of TEs dynamics on AD. This model can be used to formulate a TE dosing scheme or a reduced version of this can be used as a model predictive control of TE dosing in AD. At this stage, with unavailability of proper experimental data to calibrate and validate such a model, it is necessary to individuate and define other biochemical and physicochemical processes (EPS and redox state) which can affect the speciation of TEs in anaerobic digesters. The usefulness of such a complex dynamic model based on ADM1 to predict the speciation of TEs in anaerobic digestion system may be questioned. Nevertheless, the need to implement major processes affecting of TE dynamics in a consolidated framework is crucial for the preceding steps of model development. Therefore, some features of the model are under development and some limitations and gap areas in this study are summarized below:

1. Amino acids and proteins can be considered as a source for TEs.
2. Production of soluble microbial product as a part of biochemical processes can be included.
3. Incorporating a separate module to define and predict binding site density by considering surface complexation models.
4. Information on microbial community composition and abundance in presence of TEs can be incorporated.

Future developments include a global sensitivity analysis to individuate the most significant parameters; model calibration and validation by using ad hoc experimental data.

## Methods: model development and simulation

### Mathematical model structure

**Figure 7:**
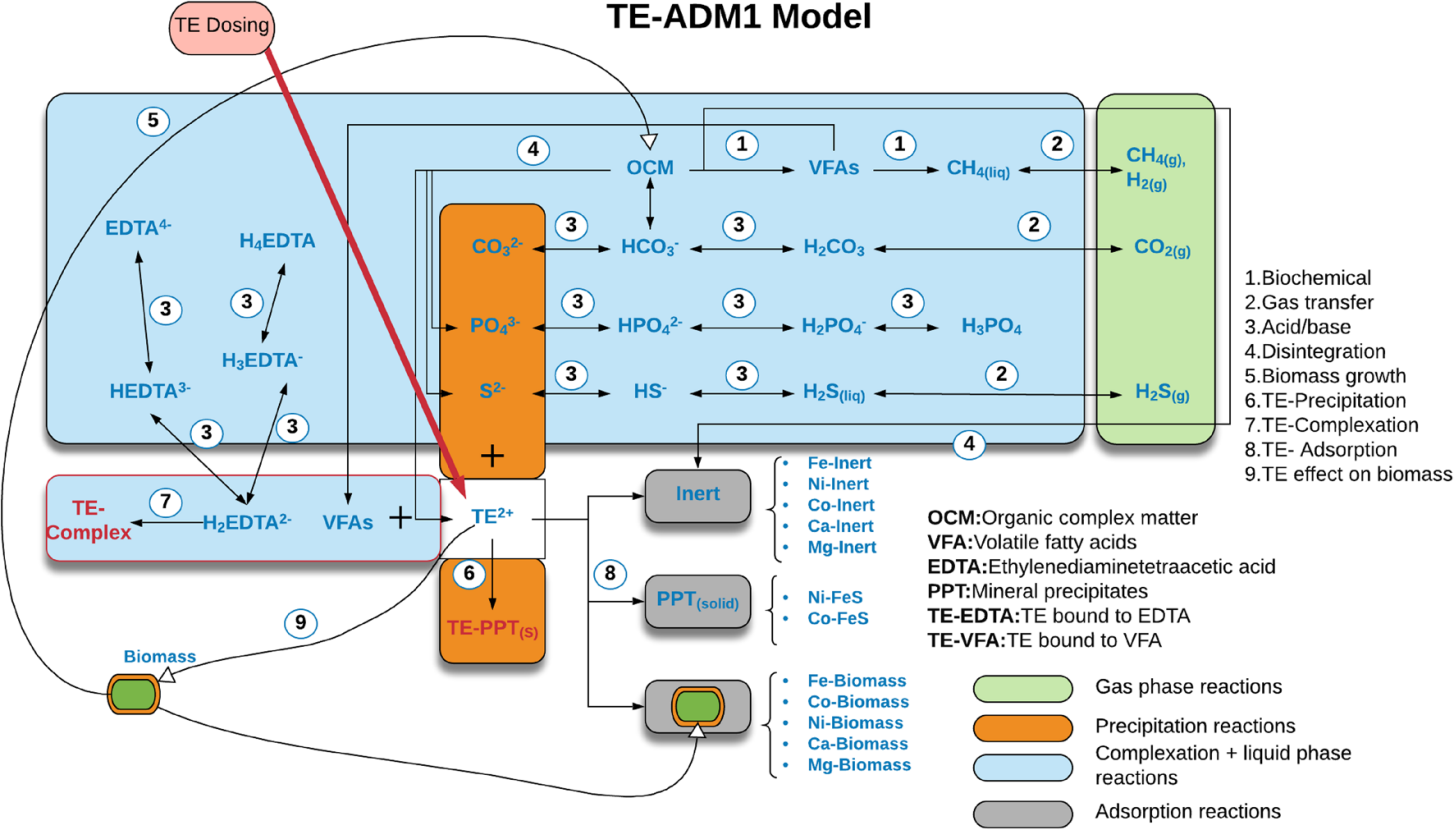
The TE-ADM1 model. Created in Lucidchart (www.lucidchart.com).

The proposed model simulates the dynamics of TEs during AD (Fig. 7). The 3-phase (solid–liquid–gas) AD model, which is based on mass conservation principle, considers a full kinetic framework to incorporate processes responsible for speciation of TEs. Speciation reactions occurring in the liquid–solid phase are central to the TE adsorption and they can be classified in three groups: adsorption on biomass, adsorption on inerts and adsorption on TE precipitates (i.e. FeS). Apart from adsorption reactions, protonation/deprotonation, TE precipitation/dissolution and TE-complexation with VFAs and EDTA have been considered^37^. Biochemical degradation of organic complex matter in the liquid phase has been considered to produce a gas mixture of H_2_, CH_4_, CO_2_ and H_2_S.

Overall, the proposed model tracks the dynamics of the state variables which represent the components of the proposed AD model. For each component considered in the liquid, gas and solid phase the general mass balance can be written as:

1 $$\begin{aligned} \frac{{{\text{dV}}_{{{\text{liq}}}} {\text{S}}_{{\text{i}}} }}{{{\text{dt}}}} & = {\text{q}}_{{{\text{in}}}} {\text{S}}_{{{\text{in}},{\text{i}}}} - {\text{q}}_{{{\text{out}}}} {\text{S}}_{{\text{i}}} + {\text{V}}_{{{\text{liq}}}} \left( {\mathop \sum \limits_{j = 1}^{{{\text{m}}_{5} }} {\updelta }_{{{\text{i}},{\text{j}}}} {\uprho }_{{{\text{A}},{\text{j}}}} \left( {{\text{t}},{\mathbf{S}}} \right) - {\uprho }_{{{\text{T}},{\text{i}}}} \left( {{\text{t}},{\mathbf{S}},{\mathbf{S}}_{{{\mathbf{gas}}}} } \right)} \right) \\ & \quad + {\text{ V}}_{{{\text{liq}}}} \left( {\mathop \sum \limits_{{{\text{j}} = 1}}^{{{\text{m}}_{1} }} {\upalpha }_{{{\text{i}},{\text{j}}}} {\uprho }_{{{\text{bio}},{\text{j}}}} \left( {{\text{t}},{\mathbf{S}},{\mathbf{X}}} \right) + \mathop \sum \limits_{{{\text{j}} = 1}}^{{{\text{m}}_{2} }} {\upbeta }_{{{\text{i}},{\text{j}}}} {\uprho }_{{{\text{cmplx}},{\text{j}}}} \left( {{\text{t}},{\mathbf{S}}} \right)} \right) \\ & \quad + {\text{V}}_{{{\text{liq}}}} \left( {\mathop \sum \limits_{{{\text{j}} = 1}}^{{{\text{m}}_{3} }} {\upgamma }_{{{\text{i}},{\text{j}}}} {\uprho }_{{{\text{prec}},{\text{j}}}} \left( {{\text{t}},{\mathbf{S}},{\mathbf{X}}_{{\mathbf{P}}} } \right) + \mathop \sum \limits_{{{\text{j}} = 1}}^{{{\text{m}}_{3} }} {\upgamma }_{{{\text{i}},{\text{j}}}} {\uprho }_{{{\text{dissol}},{\text{j}}}} \left( {{\text{t}},{\mathbf{S}},{\mathbf{X}}_{{\mathbf{P}}} } \right) + \mathop \sum \limits_{{{\text{j}} = 1}}^{{{\text{m}}_{4} }} {\upvarepsilon }_{{{\text{i}},{\text{j}}}} {\uprho }_{{{\text{s}},{\text{j}}}} \left( {{\text{t}},{\mathbf{S}},{\mathbf{X}}_{{{\mathbf{ads}}}} ,{\overline{\mathbf{X}}}_{{{\mathbf{ads}}}} } \right)} \right), \\ & {\text{i}} = 1, \ldots { },{\text{ n}}_{1} { },\quad {\text{t}} > 0, \\ \end{aligned}$$

2 $$\begin{aligned} & \frac{{{\text{dV}}_{{{\text{liq}}}} {\text{X}}_{{\text{i}}} }}{{{\text{dt}}}} = {\text{q}}_{{{\text{in}}}} {\text{X}}_{{{\text{in}},{\text{i}}}} - {\text{q}}_{{{\text{out}}}} {\text{X}}_{{\text{i}}} + {\text{ V}}_{{{\text{liq}}}} \left( {\mathop \sum \limits_{{{\text{j}} = 1}}^{{{\text{m}}_{1} }} {\upalpha }_{{{\text{i}},{\text{j}}}} {\uprho }_{{{\text{bio}},{\text{j}}}} \left( {{\text{t}},{\mathbf{S}},{\mathbf{X}}} \right){ }} \right), \\ & {\text{i}} = {\text{n}}_{1} + 1,{ } \ldots ,{\text{ n}}_{2} ,{ }\quad {\text{t}} > 0, \\ \end{aligned}$$

3 $$\begin{aligned} & \frac{{{\text{dS}}_{{{\text{gas}},{\text{i}}}} }}{{{\text{dt}}}} = - \frac{{{\text{S}}_{{{\text{gas}},{\text{i}}}} {\text{q}}_{{{\text{gas}}}} }}{{{\text{V}}_{{{\text{gas}}}} }} + \frac{{{\text{V}}_{{{\text{liq}}}} }}{{{\text{V}}_{{{\text{gas}}}} }}{\uprho }_{{{\text{T}},{\text{i}}}} \left( {{\text{t}},{\mathbf{S}},{\mathbf{S}}_{{{\mathbf{gas}}}} } \right),{ } \\ & {\text{i}} = 1,{ } \ldots ,{\text{n}}_{1} ,{ }\quad {\text{t}} > 0, \\ \end{aligned}$$

4 $$\begin{aligned} & \frac{{{\text{dV}}_{{{\text{liq}}}} {\text{X}}_{{{\text{p}},{\text{i}}}} }}{{{\text{dt}}}} = {\text{q}}_{{{\text{in}}}} {\text{X}}_{{\text{p}}}^{{{\text{in}}}} - {\text{q}}_{{{\text{out}}}} {\text{X}}_{{{\text{p}},{\text{i}}}} + {\text{ V}}_{{{\text{liq}}}} \left( {\overline{\alpha }_{i} {\uprho }_{{{\text{prec}},{\text{i}}}} \left( {{\text{t}},{\mathbf{S}},{\mathbf{X}}_{{\mathbf{P}}} } \right) + {\overline{\beta }}_{i} {\uprho }_{{{\text{dissol}},{\text{i}}}} \left( {{\text{t}},{\mathbf{S}},{\mathbf{X}}_{{\mathbf{p}}} } \right)} \right), \\ & {\text{i}} = 1,{ } \ldots ,{\text{m}}_{3} ,{ }\quad {\text{t}} > 0, \\ \end{aligned}$$

where: ${\text{n}}_{1}$ is the number of soluble components; ${\text{n}}_{2} - {\text{n}}_{1}$ is the number of particulate components, including complex organic matter and inert; ${\text{n}}_{3} = {\text{n}}_{2} - {\text{n}}_{1} + {\text{m}}_{3}$ is the number of adsorbing components, including microbial species, inerts and precipitates; ${\text{m}}_{1}$ is the number of biochemical processes considered; ${\text{m}}_{2}$ is the number of complexation processes considered; ${\text{m}}_{3}$ is the number of precipitation/dissolution processes considered; ${\text{m}}_{4}$ is the number of adsorption/desorption processes considered; ${\text{m}}_{5}$ is the number of acid–base reactions considered; ${\upalpha }_{{{\text{i}},{\text{j}}}}$ is the stoichiometric coefficient of the *i*th species on biochemical process j; ${\upbeta }_{{{\text{i}},{\text{j}}}}$ is the stoichiometric coefficient of the *i*th species on complexation process j; ${\upgamma }_{{{\text{i}},{\text{j}}}}$ is the stoichiometric coefficient of the *i*th species on precipitation/dissolution process j; ${\updelta }_{{{\text{i}},{\text{j}}}}$ is the stoichiometric coefficient of the *i*th species on acid–base reaction j; ${\upvarepsilon }_{{{\text{i}},{\text{j}}}}$ is the stoichiometric coefficient of the *i*th species on adsorption/desorption reaction j; ${\overline{\alpha }}_{i}$ and ${\overline{\beta }}_{i}$ are the stoichiometric coefficients for the precipitate formation and dissolution; ${\text{S}}_{{\text{i}}}$ is the *i*th soluble component, ${\mathbf{S}} = { }\left( {{\text{S}}_{1} ,{ } \ldots ,{\text{S}}_{{{\text{n}}_{1} }} } \right)$; ${\text{X}}_{{\text{i}}}$ is the *i*th particulate component, ${\mathbf{X}} = \left( {{\text{X}}_{{{\text{n}_1} + 1}} ,{ } \ldots ,{\text{X}}_{{{\text{n}}_{2} }} } \right)$; ${\text{X}}_{{{\text{p}},{\text{i}}}}$ is the *i*th precipitate, ${\mathbf{X}}_{{\mathbf{p}}} = \left( {{\text{X}}_{{{\text{p}},1}} ,{ } \ldots ,{\text{X}}_{{{\text{p}},{\text{m}}_{3} }} } \right)$; ${\text{X}}_{{{\text{ads}},{\text{i}}}}$ is the concentration of free binding sites for a specific adsorbent component, ${\mathbf{X}}_{{{\mathbf{ads}}}} = \left( {{\text{X}}_{{{\text{ads}},1}} ,{ } \ldots ,{\text{X}}_{{{\text{ads}},{\text{n}}_{3} }} } \right)$; ${\overline{\text{X}}}_{{{\text{ads}},{\text{i}}}}$ is the concentration of occupied binding sites for a specific adsorbent component, ${\overline{\mathbf{X}}}_{{{\mathbf{ads}}}} = \left( {{\overline{\text{X}}}_{{{\text{ads}},1}} ,{ } \ldots ,{\overline{\text{X}}}_{{{\text{ads}},{\text{n}}_{3} }} } \right)$; ${\text{S}}_{{{\text{gas}},{\text{i}}}}$ is the *i*th soluble gas component, ${\mathbf{S}}_{{{\mathbf{gas}}}} = \left( {{\text{S}}_{{{\text{gas}},{\text{1}}}} ,{ } \ldots ,{\text{S}}_{{{\text{gas}},{\text{n1}} }} } \right)$; ${\uprho }_{{{\text{A}},{\text{j}}}} \left( {{\text{t}},{\mathbf{S}}} \right)$ is the process rate for the *j*th acid base reaction; ${\uprho }_{{{\text{T}},{\text{i}}}} \left( {{\text{t}},{\mathbf{S}},{\mathbf{S}}_{{{\mathbf{gas}}}} } \right)$ is the gas transfer process rate for the *i*th soluble component; ${\uprho }_{{{\text{bio}},{\text{j}}}} \left( {{\text{t}},{\mathbf{S}},{\mathbf{X}}} \right)$ is the rate for the *j*th biochemical process; ${\uprho }_{{{\text{complx}},{\text{j}}}} \left( {{\text{t}},{\mathbf{S}},{\mathbf{X}}} \right)$ is the rate of the *j*th complexation process; ${\uprho }_{{{\text{prec}},{\text{j}}}} \left( {{\text{t}},{\mathbf{S}},{\mathbf{X}}_{{\varvec{p}}} } \right)$ is the rate for the *j*th precipitation process; ${\uprho }_{{{\text{dissol}},{\text{j}}}} \left( {{\text{t}},{\mathbf{S}},{\mathbf{X}}_{{\varvec{p}}} } \right)$ is the rate for the *j*th dissolution process; ${\uprho }_{{{\text{s}},{\text{j}}}} \left( {{\text{t}},{\mathbf{S}},{\mathbf{X}}_{{{\mathbf{ads}}}} ,{\overline{\mathbf{X}}}_{{{\mathbf{ads}}}} } \right)$ is the rate for the *j*th sorption/desorption process; ${\text{V}}_{{{\text{liq}}}}$ and ${\text{V}}_{{{\text{gas}}}}$ are the liquid and gas volume of the reactor; ${\text{q}}_{{{\text{gas}}}}$ is the gas flow rate.

### Biochemical fate of TE during AD

During AD, microorganisms are able to uptake a limited amount of TEs available in the bulk phase. However, the original ADM1 does not consider TEs as state variables by altogether neglecting any inorganic component other than ammonium. Starting with complex organic matter as the first reactant/component, AD is considered to have 5 stages of biochemical reactions: disintegration and hydrolysis which constitute extracellular processes; acidogenesis, acetogenesis and methanogenesis as intracellular processes. The organic complex matter is assumed to be a critical source of simpler compounds, as it eventually disintegrates to form carbohydrates, proteins and lipids. The model proposed in this paper considers the release of TEs and inorganic components during the disintegration process. Phosphorus, sulfur and alkali earth elements have been set as a product of the disintegration step. Phosphorous has been supposed to be released as HPO_4_^2^, which represents the most abundant form in the pH range of 6–14. Likewise, the released sulfur has been considered in the form of HS^-^ which has been depicted as the most abundant form in the pH range of 6–12. Moreover, external dosing of TEs is also included in the model as additional TEs source. TEs bio-uptake has been linked to acetate and hydrogen uptake. The biochemical stoichiometry has been updated accordingly.

### Acid–base processes affecting TEs geochemistry

In addition to the 6 acid base reactions of the original ADM1, the model includes sulfate acid base system, phosphate acid base system and carbonate acid base system to define the effect of pH and other biochemical processes on the geochemistry of TEs. The new inorganic components include bicarbonate/carbonate, phosphoric acid/dihydrogen phosphate, dihydrogen phosphoric acid/hydrogen phosphate, hydrogenphosphoric acid/phosphate, hydrogen sulfide/bisulfide and bisulfide/sulfide acid pairs. Apart from inorganic components, acid base reactions for synthetic chelators (here EDTA) have also been added as they are involved in TEs complexation reactions. The corresponding thermodynamic values and rate coefficients for the acid base processes have been sourced from literature. Charge balance has been modified in order to consider the effect of additional components which may affect pH prediction.

### Gas transfer processes affecting TEs geochemistry

The geochemistry of TEs in liquid phase is strongly affected by the H_2_S liquid–gas transfer process. The H_2_S stripping to gas phase is regulated by the equilibrium concentration of sulfur compounds in the liquid phase. TE interaction with sulfur system during precipitation and adsorption processes drags the equilibrium in the opposite direction towards solid phase. The model also considers liquid–gas transfer processes (Eq. 3) for gaseous components formed during the biological degradation of organic complex matter, namely H_2_, CH_4_ and CO_2_.

### TE speciation during AD

Modelling the dynamic behavior of TEs during AD is challenging but is necessary to effectively represent the geochemistry of TEs in anaerobic environments. The TE interaction with other compounds (sulfur, phosphorus, carbon, chelating agents and available solid surfaces), and bio-uptake require a substantial investment in model development and verification at different stages. Likewise, TEs interactions with alkali earth elements (Ca/Mg) and other compounds need to be considered, which increase model and computational complexity. Therefore, the speciation model has been developed and tested in stages.

#### TE precipitation/dissolution model

The precipitation model^26^ integrates the ADM1 with liquid–solid precipitation reactions. To this aim, new inorganic components and new precipitation/dissolution processes have been incorporated in the ADM1 framework. These new inorganic components influence the biochemical processes by affecting the pH of the system. Although the total free TEs concentration is not directly related to pH, precipitation affects the total free TEs present in an anaerobic digester. New chemical equilibrium association/dissociation, ion pairing, and precipitation reactions have been added to the ADM1. The components of the three chemical systems (carbonate, phosphate and sulfide) react, in the liquid phase, to form precipitates in solid phase. Precipitation has been modeled kinetically by considering a second order law based on the concentration of the participating components and reflecting the important steps of: development of supersaturation, nucleation and growth. Because precipitation reactions are reversible in nature, the dissolution of formed precipitate has been considered. Such dissolution reaction takes place when the system is undersaturated for a particular species and it takes place until the system remains in this condition. The precipitation/dissolution reactions are, thus, governed by the solubility product (K_sp_) values which have been collected from literature and geochemical databases. The full expression of the precipitation/dissolution reaction rates can be found elsewhere^26^.

#### TE complexation model

The complexation module focuses on TE aqueous complexation reactions taking place in AD^37^. In this model, ADM1 has been further modified in order to simulate the TE complexation, precipitation and its effect on AD. In this regard, the biodegradation processes, including hydrolysis, acidogenesis, acetogenesis, methanogenesis, and the acid–base equilibrium reactions have been coupled to the TE complexation, precipitation and redissolution processes. The incorporation of the precipitation and complexation reactions in the physicochemical module led to the definition of new inorganic components (in addition to the precipitation model) in the ADM1 framework. In particular, the complexation of TEs with EDTA and VFAs has been considered. To this aim, EDTA protonation and deprotonation reactions as well as complexation with TEs have been implemented through first order laws. Further, complexation of TEs with VFAs has been considered in the model. The TE-VFA complexes considered in the model are the most likely to be formed in a similar multispecies geochemical environment. The complexation model follows an established and tested modelling approach^35^. For instance, the overall formation constant of a particular TE-EDTA species is set at a particular value. Consequently, the reverse rate constant is calculated from the stability constant using the following relation:

5 $$K_{{\left[ {MeEDTA} \right]^{2 - } }} = \frac{{k_{1} }}{{k_{ - 1} }}$$

Subsequently, the charge balance has been modified in order to consider the effects of new components on the pH of the system. The full expression of the complexation reaction rates can be found elsewhere^37^.

#### TE sorption/desorption model

TE sorption/desorption has been modeled using a kinetic approach. Specifically, a reversible second order kinetic model has been considered for sorption (forward) and desorption (backward) reactions. Three types of adsorption surfaces have been considered: (1) Biomass; (2) Inerts and (3) Precipitates. Concerning the mineral precipitates, FeS has been recognized as the component playing the most crucial role in sorption processes^47,48^. Inert is a major source of sorbent because it harbours many complex polymeric substances, such as lignocellulose, cellulose and various complex protein moieties. The inert is composed of both organic and inorganic material. However, in the proposed model there is no distinction made on this basis. Biomass surfaces can vary widely (carboxyl, hydroxyl, sulfhydryl and phosphoryl functional groups) among different species. To rather complicate, inter species microbial diversity may be recalled. In the proposed model, universal biomass adsorption behaviour has been adopted. It has been hypothesized that the TE bacterial surface interactions are common over all the trophic groups considered in the original ADM1. This has been supported by many studies^49,50^.

Seven biomass species, as originally formulated in the ADM1, are construed to participate in the sorption/desorption reactions with all the five metals considered (TEs: Fe, Ni and Co; alkali earth metals: Ca and Mg, Table 4). Inert have been construed to take part in sorption/desorption reactions with Fe, Ni, Co, Mg and Ca, while, only Co and Ni have been considered to participate with precipitates (FeS)^47,48^. FeS has been considered as the only mineral precipitate on which adsorption of other TEs takes place which is due to unavailability of supportive studies to incorporate other minerals as adsorbent. Thus, arriving at a system which consists of 35 sorption/desorption reactions for biomass, 5 sorption/desorption reactions for inerts and 2 for sorption/desorption reactions on FeS. The rationale here is to design and implement a mechanistic model which relies on and further extends the ADM1 framework for identifying and selecting important soluble and particulate components for describing TE dynamics.

#### Binding sites

**Figure 8:**
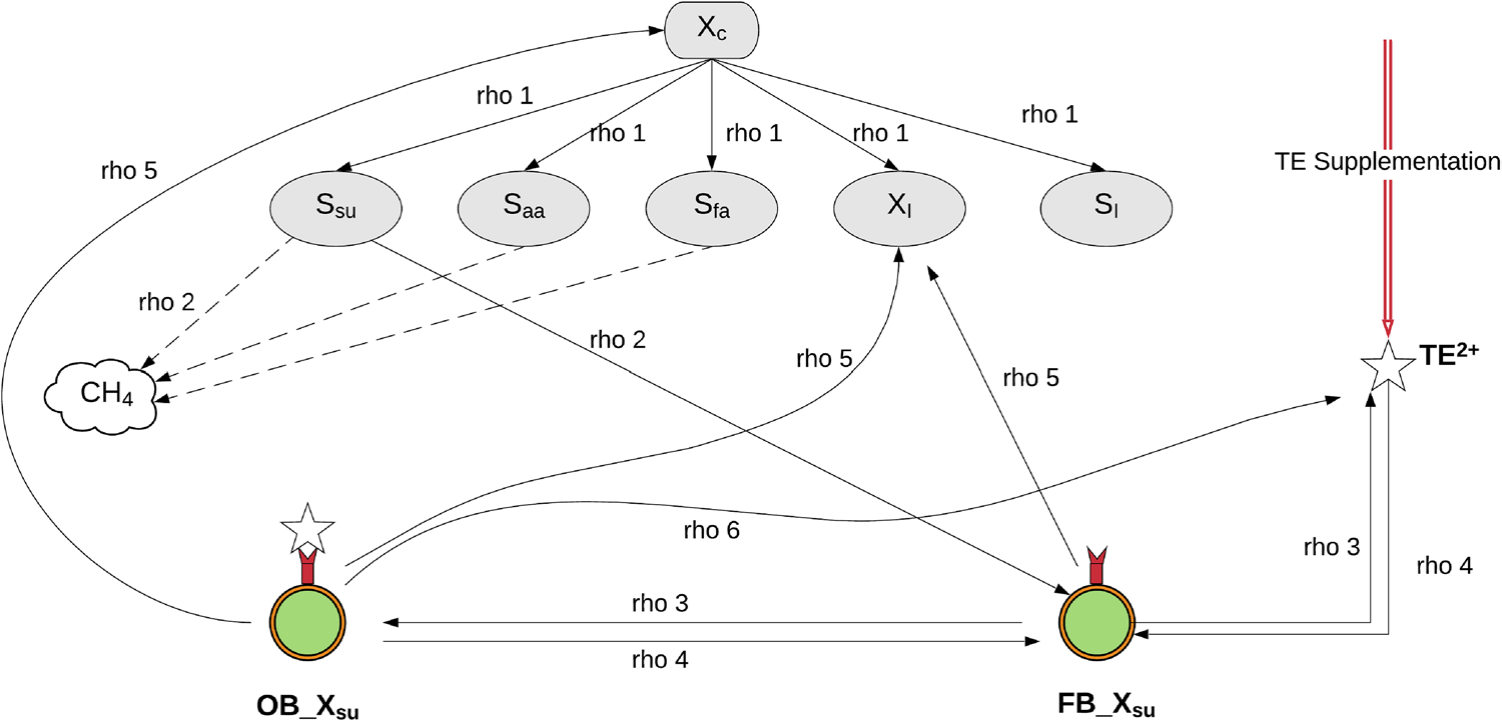
A simplified presentation of the sorption/desorption model. $rho 1$ represents the disintegration and hydrolysis step for degradation of $X_{c}$ to simpler monomers such as $S_{su}$, $S_{aa}$, $S_{fa}$, $X_{I}$ and $S_{I}$. $rho 2$ is the set of all subsequent processes in AD which lead to biomass growth and subsequent degradation of soluble metabolite into CH_4_. $rho3/rho4$ is the new adorption desorption process in ADM1 where $FB\_X_{su}$ and $OB\_X_{su}$. represent the free and binding sites concentration on biomass $X_{su}$. $rho 5$ is the biomass decay and $rho 6$ is the release of TEs concomitant to biomass decay. Created in Lucidchart (www.lucidchart.com).

The adsorption module has been conceptualized on: (1) formation of free binding sites; (2) reaction of free binding sites with TEs and alkali earth metals to form occupied binding sites-sorption reaction; (3) desorption of occupied binding sites; (4) competition among TEs for free binding sites (Fig. 8). In the sorption/desorption reactions, a pivoting role is played by the concentration of binding sites^51,52^. The latter can be found in two states: free, when the sorption reaction has not occurred yet, and occupied when the bound between the adsorbent and adsorbing components is established. The free binding site concentration is found to increase due to the formation of new sorption surfaces such as biomass, inerts and mineral precipitates. Available free TE ions react with these free binding sites leading to the formation of occupied binding sites. The extent of this conversion depends on the stability constant for the particular adsorption species considered. The adsorption process is reversible in nature. The desorption reaction leads to the release of the adsorbed TEs which decay to the bulk pool of free TEs and the formation of free binding sites. The biomass decay leads to the formation of inerts with a concomitant release of TEs adsorbed on the biomass surface. It should be noted that such decay processes are different from desorption processes. Each sorbent component is characterized by a “binding site density”. The binding site density may be defined as the moles of binding sites per unit gram of the respective surface expressed as moles/gram. Unless otherwise mentioned, a binding site density of $2 \times 10^{ - 3}$^42^ has been used as a default value for biomass and inert surfaces whereas a value of $2 \times 10^{ - 5}$ has been set for precipitate surfaces. It is based on the rational that the density of organic binding sites is higher as compared to inorganic binding sites. The competition of TEs and alkali earth metals for free binding sites has been included in the module in form of competing mass fluxes for particular free binding sites.

#### Sorption/desorption kinetics

The sorption/desorption processes are kinetically controlled and can be represented as follows:

6 $$TE^{2 + } + TES\to ^{{k_{1} }} \left( {TE \equiv TES} \right) ,$$

7 $$\left( {TE \equiv TES} \right) \to ^{{k_{ - 1} }} TE^{2 + } + TES ,$$

where, k_1_ is the $TE \equiv TES$ formation rate constant; k_−1_ is the dissociation constant. Two pairs of forward and backward reactions in (6 and 7) have equilibrium reactions and constants defined by:

8 $$TE^{2 + } + TES\mathop \leftrightarrow \limits^{{K_{TE - TES} }} \left( {TE \equiv TES} \right),$$

where, $K_{TE - TES}$ is the equilibrium constant for $TE - TES$. The rate equation for the mechanism in Eq. (8) and introduced in Eq. (1) $\rho_{s} = \rho_{ads} - \rho_{des}$ can be written as:

9a $$\rho_{ads} = K_{a/d} \cdot k_{ads} \cdot X_{{{\text{ads}}}} \cdot S_{{TE^{2 + } }} ,$$

9b $$\rho_{des} = K_{a/d} \cdot k_{des} \cdot \overline{X}_{{{\text{ads}}}} ,$$

where, $S_{{TE^{2 + } }}$, $X_{{{\text{ads}}}}$ and $\overline{X}_{{{\text{ads}}}}$ are the dynamic state variable for free TE concentration, concentration of free binding sites, concentration of occupied binding sites, respectively. $k_{ads }$ is the sorption and $k_{des}$ is the desorption kinetic rate constant. Note that $X_{ads}$ and $\overline{X}_{ads}$ constitute additional state variables of the system, whose dynamics are explicitly tracked through the sorption/desorption rate. The current model formulation considers the biomass, inerts and precipitates as having similar binding characteristics. The model does not distinguish between binding sites based on chemical characteristics. The idea here is to adapt a universal behaviour for inerts, biomass and precipitates based on binding site density which can be latter improved to incorporate specific binding characteristics, e.g. for carboxylate, phosphate and sulfhydryl surfaces.

### Effect of TEs on biochemical processes

Since TEs have been reported as micronutrients in AD systems and are constituents of co-factors in enzyme systems, their effect on biochemical processes has been explicitly taken into account by introducing an additional term $I_{{TE^{2 + } }}$ in the inhibition expressions used in ADM1^53^. The function considers both the stimulating and inhibiting action of TEs on biochemical rates depending on TE concentrations. This includes an additional non-competitive biostatic inhibition function $I_{{TE^{2 + } }}$,which keeps in track the effect of TEs on the biochemical rates and acts as a growth limiting factor. $I_{{TE^{2 + } }}$ is expressed as:

10 $$I_{{TE^{2 + } }} = \frac{{a_{1} \cdot \left( {TE^{2 + } + C^{{TE^{2 + } }} } \right) + a_{2} }}{{\left( {TE^{2 + } + C^{{TE^{2 + } }} } \right)^{2} + b_{1} \cdot \left( {TE^{2 + } + C^{{TE^{2 + } }} } \right) + b_{2} }},$$

where $a_{1} ,a_{2} ,b_{1} ,b_{2}$ are assumed constants which can been adjusted to obtain a desirable optimum dose–response function at a particular TE concentration, $TE^{2 + }$ denotes the concentration of bioavailable TEs within the bulk liquid and $C^{{TE^{2 + } }}$ is the fraction of TE which undergoes complexation reaction to form [TE-EDTA]^2,54,55^. Note that the amount of TEs adsorbed on the various surfaces present in AD is not involved in the function $I_{{TE^{2 + } }}$ as the adsorbed TEs are supposed to be unavailable for the biochemical reactions.

### Model parameters and initial conditions

An *in-silico* anaerobic batch digester of working volume 0.75 l (head space volume 0.25 l) was selected for the numerical investigations in “Scenario 1: Effect of adsorbents on TE dynamics in anaerobic digestion” to “Scenario 5: Effect of binding site density on TE dynamics in anaerobic digestion”. The temperature of the reactor was set at 35 °C. The initial conditions for the particular scenario were selected on the basis of experience and literature^9,22,53^ which have been reported in Tables 1, 2. The TEs concentration range was selected from literature^9^. The initial values for VFAs were set to zero. The initial values for ammonia and bicarbonate were adjusted at certain value to run the reactor around neutral pH. The initial amount of calcium and magnesium were taken from literature^22^. The values for the saturation constants for sorption processes were assumed in this study. The current model version assumes a single saturation constant for all the sorption/desorption reaction. The values of model parameters for biodegradation, precipitation/dissolution and complexation reactions were taken from literature^26,35,37,42,53,56^.

For model comparison with a real case data^43^, a continuous AD system which studied the effect of TEs on methane production was chosen. The initial conditions for the model simulations were selected by running the reactor for some days with the same OLR. The influent characteristics and operational parameters were obtained from literature^43^ and reported in Table 3. It was assumed that the type and number of reactions for TEs considered in the model remained unaffected with change in the mode of operation. The saturation constants for the adsorption/desorption reactions were kept at the same values as for batch simulations. The binding site density for biomass, release of TE from organic matter and uptake on the biomass were adjusted to suit the experimental behavior of methane production. All the other parameters were kept at the same values as for the batch simulations. Differently from the batch numerical investigations, the inlet and initial EDTA concentrations have been set to zero as the reactor was not augmented with any chelating agent.

**Table 1:** Variable initial concentration of complex organic matter, TEs, EDTA, Ca and Mg used in the model simulations.

RUN	Scenario 1 Variable COM (gCOD)	Scenario 2 Variable TEs (M)			Scenario 3 Variable Ca–Mg (M)	Scenario 4 Variable EDTA (M)	Scenario 5 Variable Binding density		
		Fe	Ni	Co			Biomass	Inert	Precipitate
1	1	5.0 × 10^–6^	5.0 × 10^–7^	5.0 × 10^–9^	1.0 × 10^–3^	1.0 × 10^–5^	2.0 × 10^1^	2.0 × 10^1^	2.0 × 10^–1^
2	2	9.0 × 10^–6^	9.0 × 10^–7^	9.0 × 10^–9^	1.5 × 10^–3^	2.0 × 10^–5^	2.0	2.0	2.0 × 10^–2^
3	3	3.0 × 10^–5^	3.0 × 10^–6^	3.0 × 10^–8^	2.0 × 10^–3^	3.0 × 10^–5^	2.0 × 10^–1^	2.0 × 10^–1^	2.0 × 10^–3^
4	4	7.0 × 10^–5^	7.0 × 10^–6^	7.0 × 10^–8^	2.5 × 10^–3^	4.0 × 10^–5^	2.0 × 10^–2^	2.0 × 10^–2^	2.0 × 10^–4^
5	5	1.0 × 10^–4^	1.0 × 10^–5^	1.0 × 10^–7^	3.0 × 10^–3^	5.0 × 10^–5^	2.0 × 10^–3^	2.0 × 10^–3^	2.0 × 10^–5^
6		5.0 × 10^–4^	5.0 × 10^–5^	5.0 × 10^–7^	3.5 × 10^–3^	6.0 × 10^–5^	2.0 × 10^–4^	2.0 × 10^–4^	2.0 × 10^–6^
7		9.0 × 10^–4^	9.0 × 10^–5^	9.0 × 10^–7^	4.0 × 10^–3^	7.0 × 10^–5^	2.0 × 10^–5^	2.0 × 10^–5^	2.0 × 10^–7^
8		3.0 × 10^–3^	3.0 × 10^–4^	3.0 × 10^–6^	4.5 × 10^–3^	8.0 × 10^–5^	2.0 × 10^–6^	2.0 × 10^–6^	2.0 × 10^–8^
9						9.0 × 10^–5^	2.0 × 10^–7^	2.0 × 10^–7^	
10						1.0 × 10^–4^

**Table 2:** Initial concentration of various dynamic state variables considered in the model for the batch studies.

	Variable	Scenario 1 COM	Scenario 2 Variable TEs			Scenario 3 Ca–Mg	Scenario 4 EDTA	Scenario 5 Variable Binding density			Unit
			Fe	Co	Ni			Biomass	Inert	Precipitate	
1	S_su_	0	0	0	0	0	0	0	0	0	g COD L^−1^
2	S_aa_	0	0	0	0	0	0	0	0	0	g COD L^−1^
3	S_fa_	0	0	0	0	0	0	0	0	0	g COD L^−1^
4	S_va_	0	0	0	0	0	0	0	0	0	g COD L^−1^
5	$S_{{bu^{-} }}$	0	0	0	0	0	0	0	0	0	g COD L^−1^
6	$S_{{pro^{-} }}$	0	0	0	0	0	0	0	0	0	g COD L^−1^
7	$S_{{ac^{-} }}$	0	0	0	0	0	0	0	0	0	g COD L^−1^
8	S_h2_	0	0	0	0	0	0	0	0	0	g COD L^−1^
9	S_ch4_	0	0	0	0	0	0	0	0	0	g COD L^−1^
10	S_co2_	0	0	0	0	0	0	0	0	0	g COD L^−1^
11	S_nh3_	0	0	0	0	0	0	0	0	0	g COD L^−1^
12	S_I_	0	0	0	0	0	0	0	0	0	g COD L^−1^
13	X_c_	Variable^a^	2	2	2	2	2	2	2	2	g COD L^−1^
14	X_ch_	0	0	0	0	0	0	0	0	0	g COD L^−1^
15	X_pr_	0	0	0	0	0	0	0	0	0	g COD L^−1^
16	X_li_	0	0	0	0	0	0	0	0	0	g COD L^−1^
17	X_su_	0.12	0.12	0.12	0.12	0.12	0.12	0.12	0.12	0.12	g COD L^−1^
18	X_aa_	0.12	0.12	0.12	0.12	0.12	0.12	0.12	0.12	0.12	g COD L^−1^
19	X_fa_	0.12	0.12	0.12	0.12	0.12	0.12	0.12	0.12	0.12	g COD L^−1^
20	X_c4_	0.12	0.12	0.12	0.12	0.12	0.12	0.12	0.12	0.12	g COD L^−1^
21	X_pro_	0.12	0.12	0.12	0.12	0.12	0.12	0.12	0.12	0.12	g COD L^−1^
22	X_ac_	0.12	0.12	0.12	0.12	0.12	0.12	0.12	0.12	0.12	g COD L^−1^
23	X_h2_	0.12	0.12	0.12	0.12	0.12	0.12	0.12	0.12	0.12	g COD L^−1^
24	X_I_	0	0	0	0	0	0	0	0	0	g COD L^−1^
31	S_hva_	0	0	0	0	0	0	0	0	0	g COD L^−1^
32	S_hbu_	0	0	0	0	0	0	0	0	0	g COD L^−1^
33	S_hpro_	0	0	0	0	0	0	0	0	0	g COD L^−1^
34	S_hac_	0	0	0	0	0	0	0	0	0	g COD L^−1^
35	$S_{{hco3^{-} }}$	0	0	0	0	0	0	0	0	0	M
36	$S_{{nh4^{+} }}$	0.006	0.006	0.006	0.006	0.006	0.006	0.006	0.006	0.006	M
37	$S_{{co3^{2-} }}$	0.004	0.004	0.004	0.004	0.004	0.004	0.004	0.004	0.004	M
38	$S_{{Ca^{2+} }}$	0.0025	0.0025	0.0025	0.0025	Variable^a^	0.0025	0.0025	0.0025	0.0025	M
40	$S_{{Mg^{2+} }}$	0.0025	0.0025	0.0025	0.0025	Variable^a^	0.0025	0.0025	0.0025	0.0025	M
42	$S_{{Ni^{2+} }}$	0.00001	0.00001	0.00001	Variable^a^	0.00001	0.00001	0.00001	0.00001	0.00001	M
44	$S_{{Co^{2+} }}$	0.0000001	0.0000001	Variable^a^	0.0000001	0.0000001	0.0000001	0.0000001	0.0000001	0.0000001	M
46	$S_{{Fe^{2+} }}$	0.0001	Variable^a^	0.0001	0.0001	0.0001	0.0001	0.0001	0.0001	0.0001	M
51	$S_{{po4^{3-} }}$	0.006	0.006	0.006	0.006	0.006	0.006	0.006	0.006	0.006	M
58	$S_{{hs^{-} }}$	0.001	0.001	0.001	0.001	0.001	0.001	0.001	0.001	0.001	M
71	$S_{{edta^{4-} }}$	0.0001	0.0001	0.0001	0.0001	0.0001	Variable^a^	0.0001	0.0001	0.0001	M

^a^Refer Table 1.

**Table 3:** Parameters used in model comparision with real case scenario.

Parameter	Unit	Value	Source
Total digester volume	L	4	Literature^43^
Headspace volume	L	1	Literature^43^
OLR	g VSl^−1^ day^−1^	1.45	Literature^43^
TS	%	28.1	Literature^43^
VS	% of TS	95.5	Literature^43^
TKN	% of TS	3.77	Literature^43^
Total COD	mg g^−1^	450	Literature^43^
Temperature	$^{ \circ } {\text{C}}$	37	Literature^43^
S content of FW	mol gCOD^−1^	0.0000006	In this study
P content of FW	mol gCOD^−1^	0.00000006	In this study
TE (Ni, Co, and Fe) dosed	mol gCOD^−1^	0 (starvation)	In this Study

**Table 4:** Various adsorption/desorption reactions considered in the model.

#	Description	Reaction
1	Ni-biomass adsorption/desorption	$Ni^{2 + } + X_{biomass}^{*} \leftrightarrow Ni \equiv X_{biomass}$
2	Co-biomass adsorption/desorption	$Co^{2 + } + X_{biomass} \leftrightarrow Co \equiv X_{biomass}$
3	Fe-Biomass Adsorption/Desorption	$Fe^{2 + } + X_{biomass} \leftrightarrow Fe \equiv X_{biomass}$
4	Ca-biomass adsorption/desorption	$Ca^{2 + } + X_{biomass} \leftrightarrow Fe \equiv X_{biomass}$
5	Mg-biomass adsorption/desorption	$Mg^{2 + } + X_{biomass} \leftrightarrow Fe \equiv X_{biomass}$
6	Ni-inert adsorption/desorption	$Ni^{2 + } + X_{inert} \leftrightarrow Ni \equiv X_{inert}$
7	Co-inert adsorption/desorption	$Co^{2 + } + X_{inert} \leftrightarrow Co \equiv X_{inert}$
8	Fe-inert adsorption/desorption	$Fe^{2 + } + X_{inert} \leftrightarrow Ca \equiv X_{inert}$
9	Ca-inert adsorption/desorption	$Ca^{2 + } + X_{inert} \leftrightarrow Ca \equiv X_{inert}$
10	Mg-inert adsorption/desorption	$Mg^{2 + } + X_{inert} \leftrightarrow Mg \equiv X_{inert}$
11	Ni-FeS adsorption/desorption	$Ni^{2 + } + X_{FeS} \leftrightarrow Ni \equiv X_{FeS}$
12	Co-FeS adsorption/desorption	$Co^{2 + } + X_{FeS} \leftrightarrow Co \equiv X_{FeS}$

*$X_{biomass}$ represents the different particulate biomass species considered in the original ADM1.

### Model implementation

The system of ordinary differential equations constituting the model has been implemented in an original code and has been solved using the algorithm ODE 15 s, a multistep, variable-order solver on MATLAB platform. Numerical simulations of specific scenarios have been performed to test model reliability.
